# Effects of TNF-*α* and L-Arginine Ex Vivo Treatment on Left Internal Mammary Artery (LIMA) Grafts

**DOI:** 10.3390/jcm15145716

**Published:** 2026-07-21

**Authors:** Beste Dipcin, Fatemeh Ataei, Ahmet Turan Keskintas, Gokcen Ozgun, Sevde Altuntas, Burak Onal, Lutfi Çağatay Onar, Didem Melis Oztas, Bukem Tanoren, Murat Ugurlucan

**Affiliations:** 1 Department of Biomedical Engineering, Faculty of Engineering and Natural Sciences, Acibadem Mehmet Ali Aydinlar University, Atasehir, Istanbul 34752, Turkey; 2 Acibadem University (ACU) Biomaterials A and R Center, Acibadem Mehmet Ali Aydinlar University (ACU), Atasehir, Istanbul 34752, Turkey; 3 Department of Biomedical Engineering, Graduate School of Natural and Applied Sciences, Acibadem Mehmet Ali Aydinlar University, Atasehir, Istanbul 34752, Turkey; ataeifaa@gmail.com; 4 Department of Molecular Biology and Genetics, Acibadem Mehmet Ali Aydinlar University, Atasehir, Istanbul 34752, Turkey; ahmet.keskintas@live.acibadem.edu.tr; 5 Experimental Medicine Research and Application Center, Validebag Research Park, University of Health Sciences, Uskudar, Istanbul 34668, Turkey; 6 Department of Medical Biotechnology, Graduate School of Health Sciences, Acibadem Mehmet Ali Aydinlar University, Atasehir, Istanbul 34752, Turkey; 7 Department of Tissue Engineering, University of Health Sciences, Uskudar, Istanbul 34668, Turkey; 8 Department of Pharmacology, Faculty of Medicine, Biruni University, Istanbul 34015, Turkey; 9 Department of Cardiovascular Surgery, Republic of Turkey Ministry of Health, Dr. Ismail Fehmi Cumalioglu City Hospital, Tekirdag 59020, Turkey; cagatay00@gmail.com; 10 Department of Cardiovascular Surgery, Liv Hospital Vadistanbul, Istanbul 34015, Turkey; didemmelisoztas@gmail.com (D.M.O.);; 11 Department of Natural Sciences, Faculty of Engineering and Natural Sciences, Acibadem Mehmet Ali Aydinlar University, Atasehir, Istanbul 34752, Turkey

**Keywords:** coronary artery bypass grafting, left internal mammary artery, graft patency, TNF-*α*, L-arginine, scanning acoustic microscopy, acoustic impedance

## Abstract

**Background:** Coronary artery bypass grafting (CABG) is the standard revascularization procedure, and the left internal mammary artery (LIMA) is the most widely used conduit owing to its long-term patency and favorable impact on survival. Although fibrosis and calcification are comparatively infrequent in internal mammary artery (IMA) grafts, progressive remodeling may still compromise patency. Tumor necrosis factor-α (TNF-*α*) is a pro-inflammatory cytokine implicated in vascular remodeling and calcification, whereas L-arginine, a precursor of nitric oxide, may exert vasoprotective effects. **Aim:** To investigate the effects of ex vivo TNF-*α* and combined TNF-*α* plus L-arginine treatment on the morphology, calcification, elemental composition, and biomechanical properties of human LIMA grafts. **Patients and Methods:** LIMA graft segments from 18 male CABG patients were allocated to one control group (G0) and two ex vivo treatment groups. Grafts were assessed using hematoxylin and eosin and Masson’s Trichrome staining under brightfield microscopy, using Alizarin Red S fluorescence staining to measure calcification, and by scanning electron microscopy with energy-dispersive spectroscopy (SEM/EDS) to assess ultrastructure and elemental composition. Scanning acoustic microscopy (SAM) was used to derive acoustic impedance as a label-free index of tissue stiffness. **Results:** TNF-*α* treatment was associated with increased acoustic impedance, consistent with graft stiffening, together with greater calcification signatures, whereas combined TNF-*α* and L-arginine treatment shifted impedance toward control-like values and showed more organized tissue architectures with reduced calcification. **Conclusions:** In this preliminary ex vivo model, L-arginine attenuated TNF-*α*-associated remodeling and stiffening of LIMA grafts, suggesting a potential role in supporting graft durability; larger, statistically powered studies with molecular validation are required to confirm these findings.

## 1. Introduction

Globally, cardiovascular diseases (CVDs) rank among the foremost contributors to adult mortality [1,2], with epidemiological data consistently demonstrating a disproportionately higher burden in male populations [3]. In the Turkish population specifically, the principal cardiovascular risk factors have been identified to be hypertension, smoking and tobacco use, obesity and overweight, diabetes mellitus, and hypercholesterolemia or dyslipidemia [4,5]. Beyond these established determinants, a broader range of contributors—including environmental exposures such as air pollution and socioeconomic disadvantage, lifestyle factors such as diet and physical activity, and chronic inflammatory conditions such as psoriasis, inflammatory bowel disease, and autoimmune collagen vascular disorders—has also been implicated in CVD development. Systemic inflammatory activity, characterized by elevated circulating levels of pro-inflammatory mediators including interleukin-6 (IL-6), tumor necrosis factor-α (TNF-*α*), and C-reactive protein (CRP), is frequently documented in patients with established CVD [6].

Coronary artery bypass grafting (CABG) constitutes the reference surgical intervention for myocardial revascularization. The principal conduits employed are the left internal mammary artery (LIMA) and the saphenous vein (SV) [7,8,9,10]. While the right internal mammary artery (RIMA) has been utilized in selected cases, its reported patency data remain heterogeneous [11]. The LIMA is regarded as the criterion-standard conduit given its durable long-term patency and the survival benefit it confers relative to SV grafts after myocardial infarction; suboptimal surgical technique, however, may adversely affect patency outcomes [12,13]. Despite the overall superiority of IMA grafts, early failure rates within the first year following CABG remain between approximately 2–9% [14]. The LIMA was selected as the conduit of interest in the present study because it is the criterion-standard arterial graft with the most favorable long-term patency, and because it is routinely harvested in essentially every CABG procedure; this made surplus distal LIMA segments—which would otherwise be discarded during graft preparation—readily available for ex vivo investigation without any additional intervention. Although other arterial conduits such as the radial artery are also clinically relevant, their evaluation was beyond the scope of the present study and represents a valuable direction for future comparative work.

Histologically, IMA grafts are organized into three concentric layers: the tunica intima (TI), the tunica media (TM) that houses smooth muscle cells (SMCs), and the tunica adventitia (TA), which all surround an endothelium-lined lumen [15]. Fibrosis and calcification represent well-established pathological processes that impair CABG graft patency [10]. Although these processes occur less readily in IMA grafts than in SVGs, segments arising from the left anterior descending (LAD) or left circumflex (LCX) artery territories remain susceptible to long-term calcification [16]. Following CABG, the arterialization process may trigger endothelial damage, subsequently driving SMC infiltration and upregulation of proteoglycans and collagens (types I and III) within the graft wall [15].

Tumor necrosis factor-α (TNF-*α*) is a pleiotropic pro-inflammatory cytokine implicated in tissue damage and the amplification of inflammatory cascades, and evidence indicates that it promotes both fibrotic remodeling and calcification within arterial walls. Circulating TNF-*α* levels can increase after CABG [17,18,19]. Nitric oxide (NO), synthesized by nitric oxide synthase (NOS) enzymes, serves as a critical regulator of vascular homeostasis [20], mediating vasodilatory responses and dampening inflammatory activity within vessel walls [20]. Additionally, NO exerts a protective influence against vascular injury pathways that underlie diseases such as atherosclerosis, where progressive lipid deposition and inflammatory infiltration accumulate in the arterial intima [21,22]. Experimental evidence suggests that NO may suppress vascular calcification through interference with TGF-β signaling pathways, implicating it as a potential inhibitor of pathological mineralization [23,24,25]. As a conditionally essential amino acid and the principal biosynthetic substrate for NO production, L-arginine is therefore of considerable interest as a cardiovascular-protective compound [20,22].

Scanning electron microscopy (SEM) enables detailed morphological characterization of both synthetic materials and biological specimens including tissue sections, while energy-dispersive X-ray spectroscopy (EDS) provides complementary information regarding elemental composition [26]. Image formation in SEM relies on signal generation arising from electron–sample interactions, including secondary and backscattered electrons [26].

Alizarin Red S is an anthraquinone dye that selectively binds calcium deposits and macrocalcifications in vascular and bone tissues under ex vivo conditions, producing a characteristic red fluorescent signal at calcified sites [27,28,29].

Beyond conventional histological and compositional characterization, assessment of graft biomechanical properties is of particular relevance given that inflammation-driven remodeling and progressive calcification are known to elevate tissue stiffness, potentially precipitating graft dysfunction. Scanning acoustic microscopy (SAM) represents a well-established modality for high-resolution mapping of acoustic impedance, delivering a quantitative, label-free surrogate measure of local tissue stiffness in biological soft tissues [30]. Its integration into the analytical framework enables direct quantification of mechanical alterations in LIMA grafts that complement the qualitative structural data obtained by conventional staining methods.

**Figure 1:**
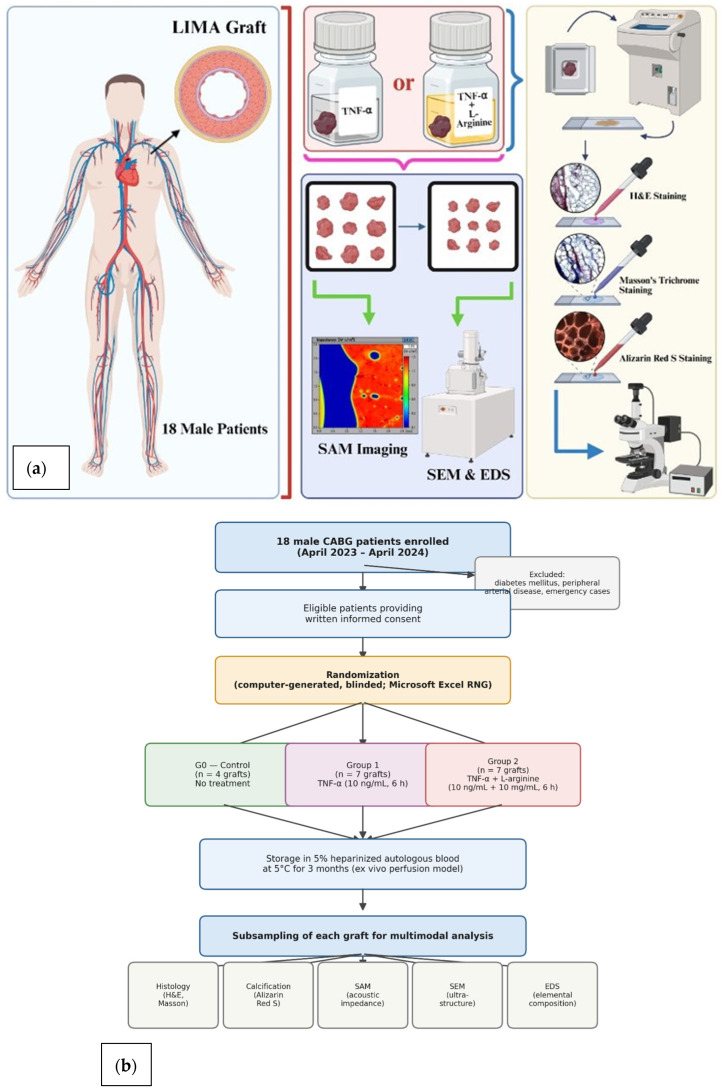
(**a**) Outline of the study (Created in BioRender. Beste Dipçin (2026). https://BioRender.com/udyamdh; accessed on 6 July 2026). (**b**) Study flow diagram showing patient enrollment, randomization, ex vivo treatment allocation, and graft subsampling for the histological, calcification, SAM, and SEM/EDS analyses. The study was conducted between April 2023 and April 2024. Abbreviations: LIMA, left internal mammary artery; CABG, coronary artery bypass grafting; TNF-*α*, tumor necrosis factor-alpha; H&E, hematoxylin and eosin; SAM, scanning acoustic microscopy; SEM, scanning electron microscopy; EDS, energy-dispersive spectroscopy; G0, control group; Group 1, TNF-*α*; Group 2, TNF-*α* + L-arginine.

TNF-*α* was chosen as the inflammatory stimulus because it is a well-characterized pro-inflammatory cytokine whose levels rise after CABG and which has been mechanistically linked to vascular remodeling and calcification, making it a physiologically relevant trigger for modeling inflammation-associated arterial injury; the ex vivo autologous-blood setup further allowed the direct, tissue-level effect of L-arginine to be examined under controlled, patient-matched conditions. The objective of the present investigation was to characterize the effects of TNF-*α* and L-arginine ex vivo conditioning on LIMA grafts through a multi-modal analytical approach encompassing Alizarin Red S fluorescence microscopy, SEM, and EDS. In parallel, SAM-derived acoustic impedance mapping was applied to provide a quantitative biomechanical assessment of treatment-related remodeling. The present preliminary work was designed to explore whether L-arginine possesses the capacity to attenuate TNF-*α*-mediated structural deterioration and thereby inform strategies aimed at maintaining LIMA graft integrity and long-term patency (Figure 1).

## 2. Materials and Methods

### 2.1. Study Population

A total of 18 consecutive male patients (*n* = 18) who underwent CABG between April 2023 and April 2024 were enrolled in this experimental study. Written informed consent was obtained from each participant following thorough explanation of the study protocol. Diabetes mellitus and peripheral arterial disease were confirmed based on documented diagnoses in the patients’ medical records; patients with either condition, as well as emergency cases, were excluded. In this study, the term “clinically stable” refers to patients free of acute coronary symptoms, hemodynamic instability, or decompensated heart failure at the time of elective surgery. Accordingly, all enrolled patients were clinically stable and eligible for elective cardiac surgery and had a history of tobacco use (active or past).

The 18 participants were distributed by computer-generated simple randomization into one control group (G0) and two experimental groups (Group 1 and Group 2). Four grafts were assigned to G0 (control), with the remaining grafts distributed between the two experimental groups; the control group (G0) functioned as an untreated reference group used to establish baseline graft characteristics, for which a smaller sample size was sufficient, whereas the larger allocation to the two experimental groups reflected the primary aim of comparing the active treatment conditions. A blinded investigator performed all randomization using a random-number generator in Microsoft Excel, thereby ensuring allocation concealment throughout the assignment process. In detail, each enrolled patient was assigned a sequential identification number, and a corresponding list of random numbers was generated in Microsoft Excel using the RAND function by an investigator who was not involved in tissue processing or imaging. Patients were then ordered by their random values and allocated to the control group (G0) or to one of the two experimental groups (Group 1 or Group 2) according to this ranking, so that group assignment was independent of patient characteristics. For the age-based analyses, grafts were subsequently stratified by patient age into three predefined strata, namely 45–55, 56–65, and 66–75 years, to allow for age-related comparisons of morphology, calcification, and elemental composition. Because of the small number of samples per group (G0, *n* = 4; Group 1, *n* = 7; Group 2, *n* = 7), the data were analyzed using descriptive statistics only, and no inferential (hypothesis-testing) statistics were applied. For each treatment group, continuous variables (SAM-derived acoustic impedance and EDS-derived atomic percentages) were summarized as the group mean accompanied by the standard deviation (SD) and the observed range (minimum–maximum); individual per-sample values, together with their instrument-derived measurement uncertainties. Patient ages were summarized as individual values and as predefined strata (45–55, 56–65, and 66–75 years). Given the limited and unequal group sizes and the exploratory nature of the study, no *p*-values, confidence intervals, or other inferential measures were calculated; consequently, all between-group differences described below are reported as descriptive trends rather than as statistically tested effects. Descriptive calculations were performed in Microsoft Excel. The enrollment, randomization, and subsequent subsampling of grafts are summarized in Figure 1b.

### 2.2. Tissue Collection

During bypass surgery, the left internal thoracic artery (LIMA) was collected from each patient. Distal arterial segments discarded during graft preparation were retrieved and allocated to one control group and two experimental groups. The control group (G0; *n* = 4 grafts) received no treatment. Grafts in Group 1 were incubated with TNF-*α* (10 ng/mL) for 6 h to induce endothelial injury and were then maintained in heparinized autologous blood. Grafts in Group 2 underwent the identical endothelial-injury protocol but with L-arginine (10 mg/mL) additionally supplemented into the heparinized autologous blood solution, allowing for the assessment of the effect of L-arginine on injured endothelium. All specimens were subsequently refrigerated at 5 °C for 3 months in 5% heparinized autologous blood solution to establish an ex vivo perfusion model simulating physiological conditions and to prevent coagulation. Throughout this storage period, specimens were maintained under controlled conditions at 5 °C in sterile containers and monitored regularly to prevent contamination, thereby preserving both structural integrity and tissue viability. Upon completion of the three-month incubation, grafts were directly examined by scanning electron microscopy to characterize endothelial ultrastructure, and were subsequently fixed in 4% formaldehyde for cryosectioning and downstream histological analyses.

### 2.3. Tissue Sectioning

LIMA grafts were first macro-sectioned using a scalpel and subsequently processed by cryosectioning on a Leica CM1860-UV cryostat (Leica Biosystems, Nußloch, Germany). For embedding, graft segments were mounted on tissue holders and rapidly frozen at −30 °C, and embedded into an optimum cutting temperature (OCT) gel (Sigma-Aldrich, Bayern, Germany, Cat. #: SLCB3563). Cryosections were prepared at a standardized section thickness of 20 μm and mounted onto glass microscope slides for subsequent analyses.

### 2.4. Tissue Section Staining and Imaging and Image Analysis

For hematoxylin and eosin (H&E) staining, slides were rinsed under running tap water, then immersed in hematoxylin solution (Bio-Optica, Milan, Italy) for 4 min. After a subsequent rinse, sections were counterstained with eosin (Atom Scientific LTD, Hyde, UK, Cat. #: RRSP35/E) for 1.5 min. Slides were dehydrated through a graded ethanol series (85% and 95%, 30 s each) followed by an approximately 2 min clearing step in pure acetone (Sigma-Aldrich, Bayern, Germany, Cat. #: 179124) to achieve optical transparency. Coverslips were applied using SP15 mounting medium (Fisher Chemical, Fair Lawn, NJ, USA). Masson’s Trichrome staining was carried out using a commercial kit (HistoPlus, São Paulo, Brazil, Cat. #: HST-MTA-0100), after which coverslips were affixed with SP15 mounting medium (Fisher Chemical, Fair Lawn, NJ, USA). For Alizarin Red S staining, slides were processed under darkened conditions by incubating them with Alizarin Red S fluorescent dye (GBL, Umraniye, Istanbul, Turkey) for 4 min and making the slides transparent using pure acetone before they were covered with a coverslip using a mounting medium (Fisher Chemical, Fair Lawn, NJ, USA, Cat. #: SP15).

H&E- and Masson’s Trichrome-stained sections were visualized by brightfield microscopy (Zeiss Axio Observer). Alizarin Red S-stained sections were imaged by fluorescence microscopy (Zeiss Axio Observer) at 10× magnification using 475 nm excitation. Image processing and quantification of fluorescence intensities were performed with ZEN Pro 3.6 software. Given the limited sample size (*n* = 4 per group), formal statistical analysis of fluorescence intensity data was not conducted.

### 2.5. Scanning Acoustic Microscopy (SAM) and Acoustic Impedance Analysis

SAM measurements were conducted on LIMA graft sections using a Honda Electronics AMS-50SI acoustic microscope (Honda Electronics Co., Ltd., Toyohashi, Aichi, Japan) configured in impedance mode with an 80 MHz transducer. Distilled water served as both the acoustic coupling medium and the calibration reference (Z_ref = 1.48 MRayl). At the outset of each imaging session, a reference intensity value (I_ref) was acquired under identical instrumental conditions and applied uniformly to all specimens examined on the same day. To minimize acoustic artifacts arising from surface curvature, samples were mounted flat on a substrate submerged in a thin water film.

Acoustic images were processed in ImageJ (1.54p) (National Institutes of Health, Bethesda, MD, USA). For each specimen, three to five regions of interest (ROIs) were defined, deliberately excluding areas affected by surface irregularities or imaging artifacts. The mean gray-level intensity of each ROI (*I_s_*) was extracted, and the acoustic reflection coefficient (R) was derived as

$$R=\frac{I_{s}-I_{ref}}{I_{s}+I_{ref}}$$

Acoustic impedance of the sample ($Z_{sample}$) was then derived using

$$Z_{sample}=Z_{ref}\times \frac{1+R}{1-R}$$

Impedance data are presented with associated measurement uncertainty derived from intra-ROI intensity variability. Between-group comparisons were performed to evaluate mechanical property differences attributable to the respective ex vivo treatments.

### 2.6. Scanning Electron Microscopy and Energy Dispersive Spectroscopy

SEM and EDS were applied in tandem to obtain morphological and elemental characterization of the graft tissues, respectively.

For each experimental group, two IMA grafts were selected on the basis of patient age (one younger and one older donor; *n* = 2 per group). Graft fragments not exceeding 0.5 cm were trimmed and subjected to critical point drying with carbon dioxide using acetone as the transitional solvent in a Leica© EM CPD300 Critical Point Dryer device. Drying was completed at 40 °C without compromising section integrity, thereby preventing morphological distortion and rendering specimens compatible with high-vacuum SEM imaging.

Dried specimens were adhered to aluminum stubs via carbon tape for SEM (Thermo Scientific™ Quattro ESEM, Waltham, MA, USA) and sputter-coated with gold (40 nm, 3 deposition cycles) using a Leica EM ACE 200 instrument prior to imaging. Elemental analysis was performed with an EDAX EDS detector operated through APEX software version 1.3 (EDAX, Inc., Mahwah, NJ, USA). Before measurements, the system was auto-calibrated using aluminum (Al) and copper (Cu) reference standards. EDS spectra were acquired from representative areas at 1000× magnification to determine mean atomic percentages. Quantified elements comprised carbon (C), nitrogen (N), oxygen (O), sodium (Na), sulfur (S), and calcium (Ca). Due to the limited number of measurements per specimen (*n* = 3 per tissue), formal statistical evaluation of elemental composition data was not performed.

## 3. Results

Table 1 presents the complete list of LIMA grafts included in this study, together with the corresponding patient ages and assigned ex vivo treatment conditions.

**Figure 2:**
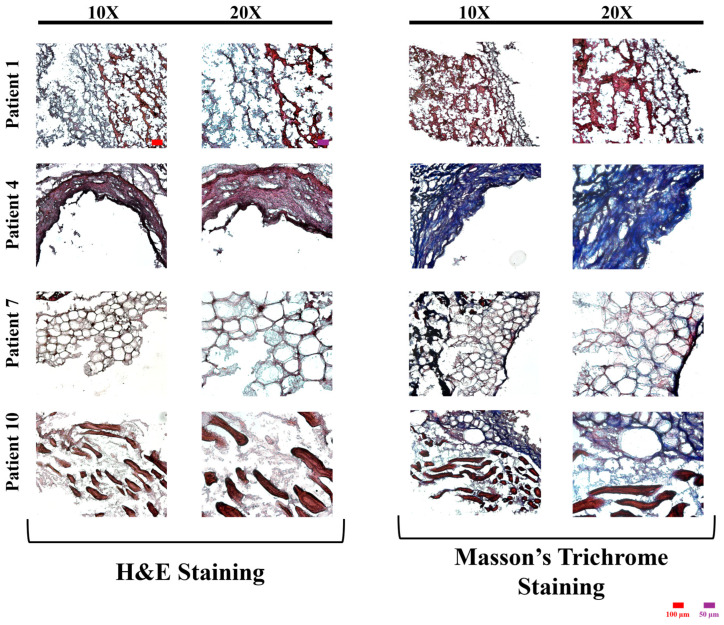
Images (10× and 20×) of Hematoxylin & Eosin (H&E)- and Masson’s Trichrome-Stained Control Group LIMA Grafts. Scale bar = 100 μm and 50 μm for 10× and 20×, respectively.

**Figure 3:**
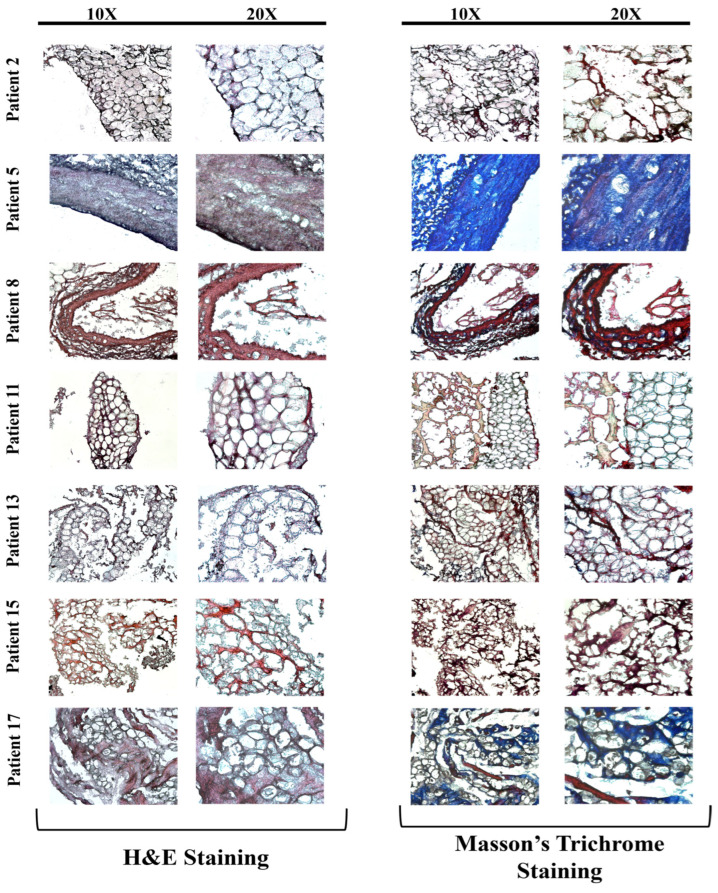
Images (10× and 20×) of Hematoxylin & Eosin (H&E)- and Masson’s Trichrome-Stained TNF-α-Treated Group LIMA Grafts. Scale bar = 100 μm and 50 μm for 10× and 20×, respectively.

**Figure 4:**
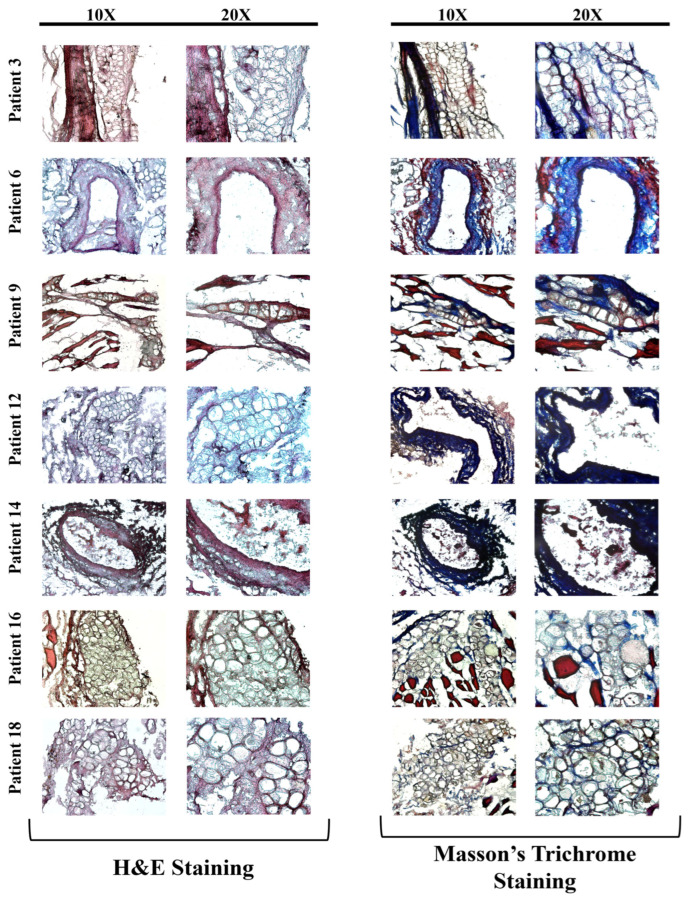
Images (10× and 20×) of Hematoxylin & Eosin (H&E)- and Masson’s Trichrome-Stained TNF-α & L-arginine-Treated Group LIMA Grafts. Scale bar = 100 μm and 50 μm for 10× and 20×, respectively.

Histological images of H&E- and Masson’s Trichrome-stained sections at two magnifications are shown in Figure 2 (control group), Figure 3 (TNF-*α*), and Figure 4 (TNF-*α* and L-arginine group).

**Figure 5:**
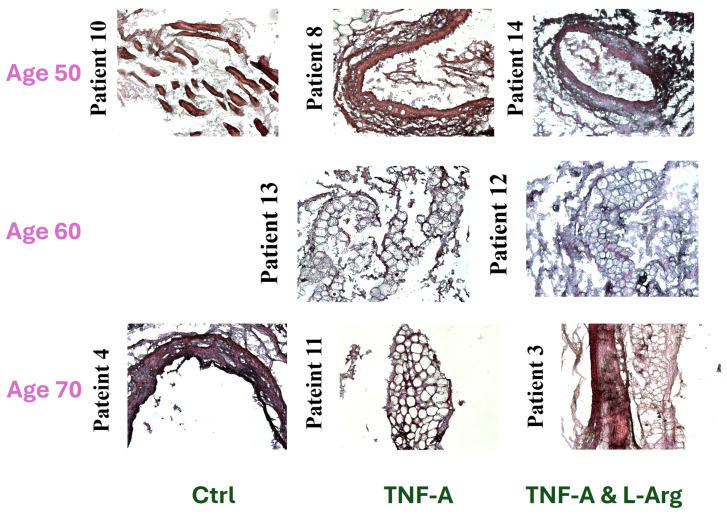
Images (10×) of H&E-Stained Control, TNF-α-Treated, and TNF-α & L-arginine-Treated LIMA Grafts from patients in three age strata (approximately 45–55, 56–65, and 66–75 years). Scale bar = 100 μm for 10×.

**Figure 6:**
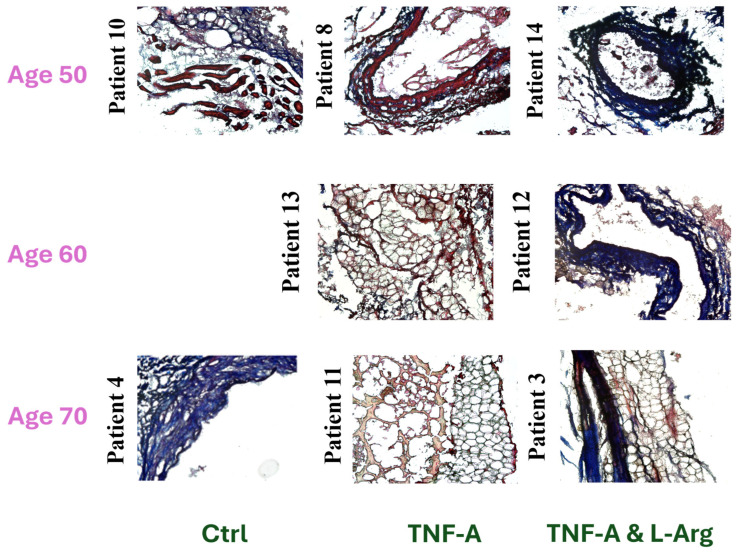
Images (10×) of Masson’s Trichrome Stained-Control, TNF-α-Treated, and TNF-α & L-arginine-Treated LIMA Grafts from patients in three age strata (approximately 45–55, 56–65, and 66–75 years). Scale bar = 100 μm for 10×.

The images of the H&E- and Masson’s Trichrome-stained LIMA grafts of the treated groups are presented in Figure 5 and Figure 6, stratified by treatment type and patient age.

**Figure 7:**
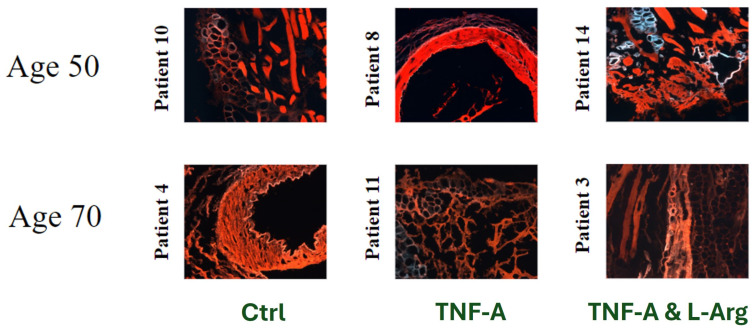
Images (10×) of Alizarin Red S-Stained Control, TNF-α-Treated, and TNF-α & L-arginine-Treated LIMA Grafts from patients in two age strata (approximately 45–55 and 66–75 years). Scale bar = 100 μm for 10×.

Fluorescence images of Alizarin Red S-stained LIMA grafts, stratified by age and treatment, are displayed in Figure 7. Because no control-group specimens were available from patients in the sixth decade of life, Alizarin Red S staining was not performed for the 60-year subgroup.

To complement the histological, fluorescence-based, and SEM/EDS findings, SAM was employed to generate quantitative acoustic impedance maps as a label-free surrogate of tissue mechanical properties. Representative impedance maps illustrating treatment-related contrast differences are displayed below, and individual patient impedance values (Z) with associated measurement uncertainties are compiled in Table 2.

**Figure 8:**
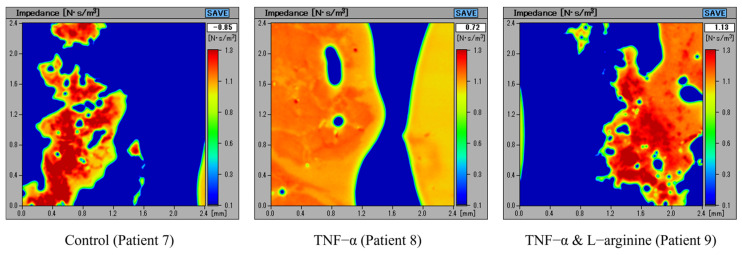
Representative SAM acoustic impedance maps of LIMA grafts by treatment group. Impedance contrast reflects relative stiffness, where higher impedance corresponds to greater local stiffness.

**Figure 9:**
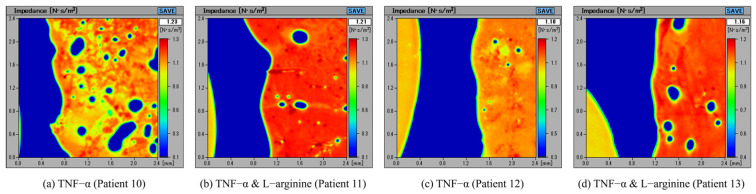
Representative paired SAM acoustic impedance maps comparing TNF-α- and TNF-α & L-Arginine-treated LIMA grafts. (**a**) TNF-α (Patient 10), (**b**) TNF-α & L-Arginine (Patient 11), (**c**) TNF-α (Patient 12), (**d**) TNF-α & L-Arginine (Patient 13). Impedance contrast reflects relative stiffness, and higher impedance corresponds to greater local stiffness.

In the representative example shown in Figure 8, TNF-*α* treatment resulted in higher impedance contrast relative to the control condition, whereas TNF-*α* and L-Arginine treatment resulted in reduced impedance contrast compared with TNF-*α* alone, consistent with attenuation of treatment-associated stiffening (Figure 9).

**Figure 10:**
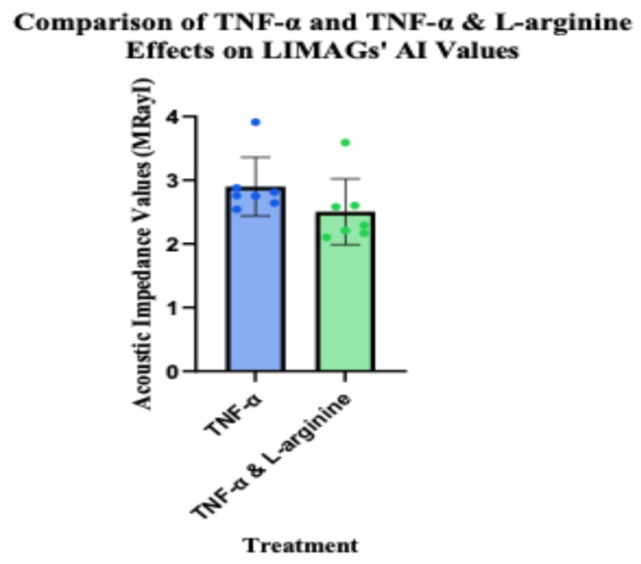
Age-independent comparison of SAM-derived acoustic impedance values in LIMA grafts from TNF-α and TNF-α & L-arginine treatment groups. Bars represent group mean acoustic impedance values.

Figure 10 presents an age-independent group comparison obtained by pooling impedance data across all patients, highlighting the systematic difference in Z attributable to treatment allocation between the TNF-*α* and TNF-*α* and L-arginine treatment groups. As shown in Figure 10, the mean acoustic impedance of the TNF-*α* group was higher than that of the TNF-*α* + L-arginine group, consistent with greater treatment-associated stiffening in the absence of L-arginine. Specifically, the mean acoustic impedance was 2.26 MRayl (SD: 0.22; range: 2.04–2.56; *n* = 4) in the control group (G0), 2.90 MRayl (SD: 0.46; range: 2.54–3.91; *n* = 7) in the TNF-*α* group, and 2.51 MRayl (SD: 0.52; range: 2.10–3.59; *n* = 7) in the TNF-*α* + L-arginine group. The TNF-*α* group thus showed the highest mean impedance, whereas co-treatment with L-arginine returned the group mean toward the control range, supporting a descriptive trend of L-arginine–associated attenuation of graft stiffening. As noted in the Methods, these summary values are descriptive; owing to the small, unequal group sizes, no statistical significance testing was performed. As this was a preliminary study and no inferential statistical testing was performed, these values are presented as group means only, without formal confidence intervals or significance testing.

At the individual patient level, the impedance values compiled in Table 2 corroborate the patterns visible in the representative maps: elevated acoustic impedance was consistently recorded in TNF-*α*-treated LIMA grafts compared with untreated controls, while the TNF-*α* and L-arginine group showed lower impedance relative to TNF-*α* alone, a pattern consistent with attenuation of inflammation-driven mechanical deterioration.

**Figure 11:**
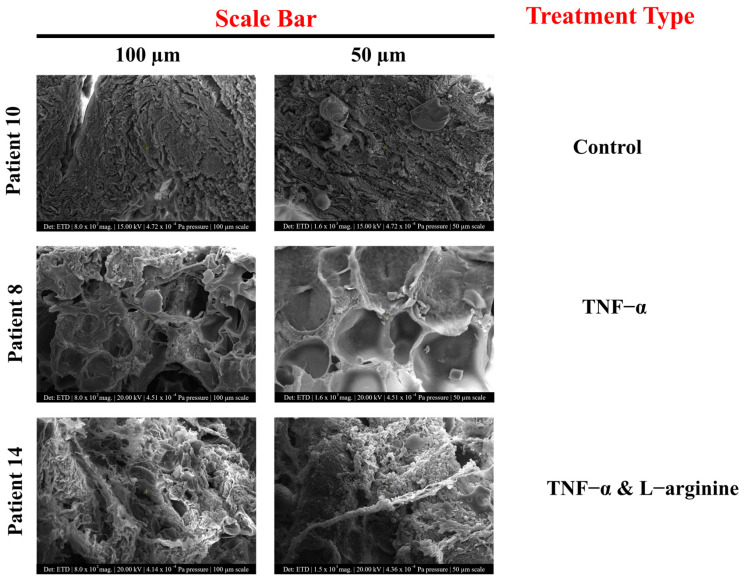
SEM Images of Control, TNF-α-Treated, and TNF-α & L-arginine-Treated LIMA Grafts from patients in the younger age stratum (approximately 45–55 years). Scale bar = 100 μm and 50 μm.

**Figure 12:**
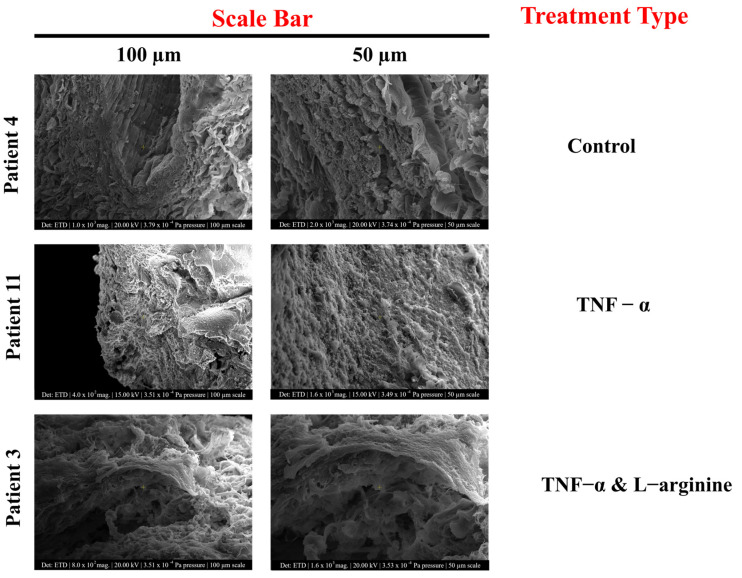
SEM Images of Control, TNF-α-Treated, and TNF-α & L-arginine-Treated LIMA Grafts from patients in the older age stratum (approximately 66–75 years). Scale bar = 100 μm and 50 μm.

To examine age-related and treatment-dependent morphological differences, SEM imaging was performed at two magnifications on grafts from patients in the younger age stratum (approximately 45–55 years; Figure 11) and the older age stratum (approximately 66–75 years; Figure 12).

**Figure 13:**
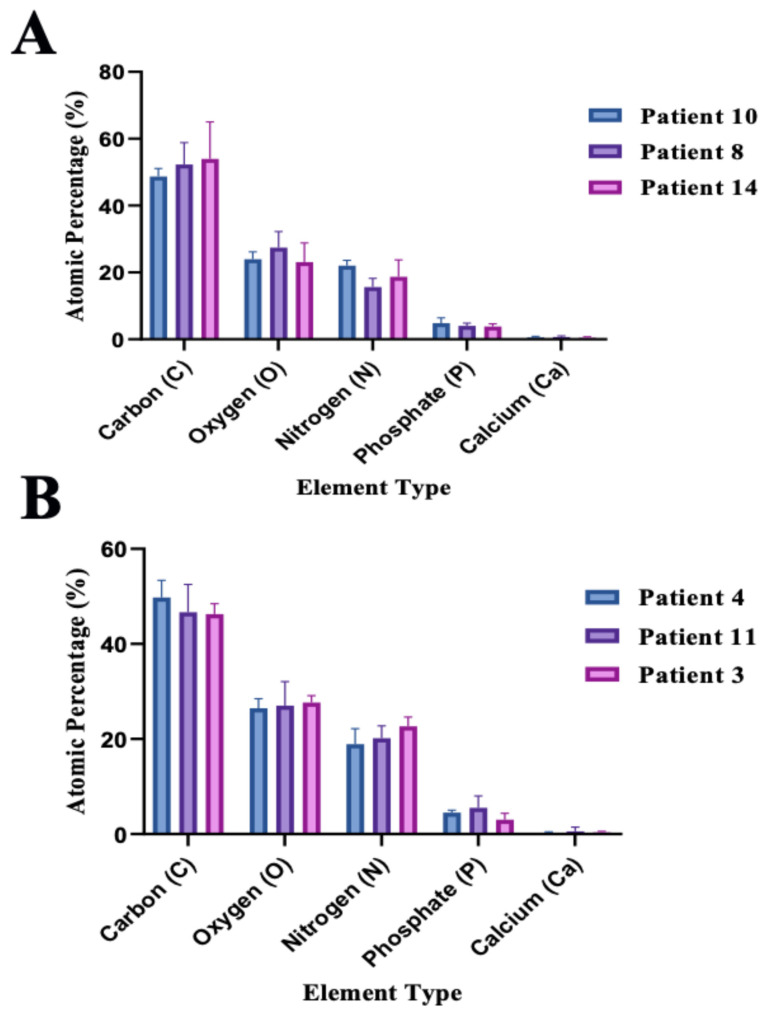
Atomic percentage (%) of carbon, oxygen, nitrogen, phosphate, and calcium elements in LIMAGs from patients of different ages and subjected to different treatments: (**A**) younger stratum (approximately 45–55 years), (**B**) older stratum (approximately 66–75 years); Blue: Control, Purple: TNF-α treatment, Pink: TNF-α & L-arginine treatment.

**Figure 14:**
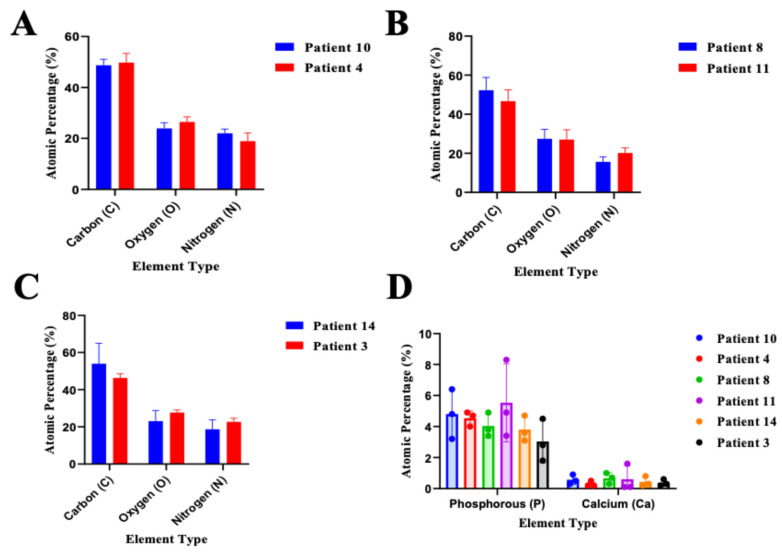
EDS analysis results for atomic percentage (%) and comparison of different elements in LIMAGs from patients of different ages and subjected to different treatments. (**A**) Control group, (**B**) TNF-α-treated group, (**C**) TNF-α & L-arginine-treated group, (**D**) P and Ca atomic percentages (%).

Table 3 presents the complete EDS dataset, listing the atomic percentages of all measured elements with associated uncertainties for each graft specimen. Age- and treatment-stratified comparisons of C, N, and O atomic percentages for the younger (approximately 45–55 years) and older (approximately 66–75 years) age strata are shown in Figure 13A,B, respectively. Treatment-group-specific elemental profiles for C, N, and O are presented for the control arm in Figure 14A, the TNF-*α* group in Figure 14B, and the TNF-*α* and L-arginine group in Figure 14C. Cross-group comparison of phosphorus (P) and calcium (Ca) atomic percentages stratified by age and treatment is shown in Figure 14D.

## 4. Discussion

The present study was designed to examine the extent to which TNF-*α* alone, or TNF-*α* in combination with L-arginine, modulates the morphological, histological, calcification-related, and elemental features of LIMA grafts obtained from male patients, and to explore the interaction of these treatment effects with patient age.

When grafts were co-treated with L-arginine alongside TNF-*α*, a more compact and well-organized vascular architecture was observed relative to grafts exposed to TNF-*α* alone when H&E-stained sections were compared across groups, suggesting that L-arginine may facilitate tissue structural reorganization (Figure 2, Figure 3 and Figure 4).

Masson’s Trichrome staining suggested a tendency toward greater vascular fibrosis with increasing patient age across treatment conditions (Figure 2, Figure 3 and Figure 4). While vascular fibrosis is recognized to be associated with factors such as tissue aging, oxidative stress, genetic predisposition, and dietary salt intake in the literature [31], these factors were not assessed in the present study, and our observations are therefore descriptive rather than causal.

A comparison of the representative images from Patient 4 (72 years) and Patient 10 (48 years) in Figure 2 shows more prominent fibrosis in the younger individual. Because individual-level data on lifestyle, genetic background, and other relevant clinical variables were not collected for these patients, this discordance cannot be mechanistically explained from the present dataset; it nonetheless illustrates that fibrosis in these grafts is multifactorial and not determined by chronological age alone [31,32].

Analysis of age-stratified sections further demonstrated that advancing age was associated with greater degrees of vascular fibrosis (Figure 5 and Figure 6), corroborating prior reports. Such fibrotic remodeling is recognized to impair graft patency and to contribute to stiffening of the vessel wall alongside extracellular matrix (ECM) degradation [17,31,33].

Of note, ex vivo L-arginine co-administration alongside TNF-*α* augmented the collagen content and reduced visible fibrosis, as evidenced in Figure 6, regardless of patient age, indicating that it may hold promise as a therapeutic strategy for limiting fibrotic progression and enhancing LIMA graft patency [20,22].

Six tissue sections per group were stained with Alizarin Red S to evaluate the impact of age and treatment on graft calcification, exploiting this stain’s established utility for detecting macrocalcifications [27]. Quantification of calcified deposits is recognized as a meaningful indicator of conduit patency and quality [10]. Comparative image analysis demonstrated that TNF-*α*-treated grafts exhibited a greater extent of calcium-positive (red) regions relative to both untreated controls and grafts treated with the TNF-*α* combination (Figure 7). This pattern implies that increasing age, in conjunction with TNF-*α* exposure, promotes calcification—a conclusion further supported by the elevated atomic calcium percentages recorded in EDS measurements (Table 3).

The acoustic impedance (Z) values obtained by SAM provided a quantitative biomechanical dimension to the analysis, complementing the qualitative structural information derived from staining-based and elemental methods. Because Z is sensitive to local microstructural features such as extracellular matrix organization and the degree of mineralization, it enables direct quantification of mechanical changes rather than their indirect inference from morphological observations alone. In the present cohort, TNF-*α* treatment yielded systematically higher impedance values compared with untreated controls (Table 2), which aligns with inflammation-induced tissue stiffening. Crucially, the TNF-*α* and L-arginine group showed broadly attenuated impedance relative to the TNF-*α*-only group (Table 2), pointing to suppression of TNF-*α*-driven biomechanical deterioration and partial restoration of graft mechanical properties. The concordance of this SAM-derived finding with the histological evidence of greater tissue organization under L-arginine co-treatment, and with the diminished calcification signals detected by Alizarin Red S, is notable. Given that both vascular calcification and fibrosis independently elevate tissue stiffness, the coherence across SAM impedance data, Alizarin Red S findings, and EDS elemental profiles strengthens the conclusion that L-arginine counteracts TNF-*α*-mediated remodeling cascades that underlie mechanical deterioration and threaten long-term graft patency.

SEM analysis of grafts subjected to the same ex vivo treatment but obtained from patients of differing ages revealed that tissue from patients in the fifth decade of life exhibited more ordered ultrastructural organization than that from patients in their eighth decade (Figure 11 and Figure 12), corroborating an age-associated loss of structural compactness in LIMA grafts. TNF-*α* treatment induced discernible morphological disruption independent of patient age, whereas L-arginine co-administration alongside TNF-*α* restored graft morphology toward a configuration more closely resembling that of the untreated controls, yielding substantially more organized tissue architectures than grafts treated with TNF-*α* alone, irrespective of patient age, an effect consistent with attenuation of vascular injury (Figure 11 and Figure 12) [19,22].

Among all treatment conditions, grafts from patients in the fifth decade of life exhibited comparatively higher atomic carbon percentages, a finding consistent with greater collagen abundance within the extracellular matrix (Figure 13 and Figure 14A–C) [34].

The graft from Patient 11, which received TNF-*α* treatment, exhibited the highest recorded atomic percentages of both Ca and P, a finding corroborated by the lipid-rich morphology evident on Masson’s Trichrome staining (Figure 3). Across the group, TNF-*α*-treated grafts consistently demonstrated elevated Ca and P atomic percentages, reinforcing the interpretation that heightened TNF-*α* activity within arterial tissue promotes calcification [19]. Co-administration of L-arginine was associated with increases in atomic N and O percentages, a pattern that may reflect enhanced NO biosynthesis given L-arginine’s established role as the primary NO precursor [20].

The current study was restricted to male patients, and future investigations incorporating female donors would be of considerable value. Women typically manifest cardiovascular symptoms at a later age than men, yet face higher perioperative and postoperative mortality following CABG; strategies that enhance graft patency therefore carry particular relevance for extending post-surgical survival in this population [35].

Given the recognized influence of lifestyle behaviors and hereditary factors on arterial biology, prospective studies incorporating detailed phenotypic and genetic characterization of patient cohorts would provide important mechanistic insight [32].

Molecular-level profiling, including quantification of mRNA transcripts and protein expression for type I/III collagen, proteoglycans, and additional ECM constituents, would substantially deepen our understanding of how the applied treatments influence graft patency [15].

The present findings document the modulatory potential of L-arginine when applied concurrently with TNF-*α*; nonetheless, dedicated studies measuring molecular indices of NO production, oxidative stress, and inflammatory activation will be required to fully delineate the mechanism of action of L-arginine under TNF-*α* co-treatment conditions [20,22].

Although Alizarin Red S staining and EDS analysis successfully identified macrocalcifications and elemental calcium deposits, these modalities are not sensitive to microcalcification. Future studies employing Raman spectroscopy would enable a more comprehensive characterization of the calcification spectrum across different graft types and treatment conditions [36].

TNF-*α* has been linked to increased vascular stiffness; however, its precise quantitative impact on LIMA graft mechanics warrants further investigation. SAM-based acoustic wave speed analysis in future studies could provide a more rigorous quantitative assessment of TNF-*α*-associated arterial stiffness changes [31,37].

The impact of L-arginine or TNF-*α* treatment on elemental graft composition was explored through EDS; however, the small sample size precluded formal statistical inference. Expansion of the sample cohort in future work is planned to enable statistically powered conclusions regarding treatment-driven elemental composition changes.

From a mechanistic standpoint, the divergent behavior of the TNF-*α* and TNF-*α* + L-arginine groups is consistent with the central role of the nitric oxide (NO) pathway in vascular homeostasis. TNF-*α* is known to promote endothelial activation, oxidative stress, and a pro-calcific phenotype in vascular smooth muscle cells, processes that collectively increase extracellular matrix deposition and tissue stiffness. L-arginine, as the physiological substrate for endothelial NO synthase, can enhance NO availability, which in turn exerts anti-inflammatory, anti-proliferative, and anti-calcific effects on the vessel wall. The observation that co-treatment with L-arginine shifted the mean acoustic impedance back toward control values, alongside more organized tissue architectures and reduced calcification signatures, is therefore mechanistically coherent: it suggests that restoring NO precursor availability may partially counteract the TNF-*α*-driven remodeling that compromises graft integrity. While our study was not designed to measure NO directly, this interpretation is in line with prior reports linking impaired NO bioavailability to accelerated graft degeneration and with experimental work in which L-arginine supplementation improved endothelial function.

These findings may carry translational relevance for the long-term durability of arterial conduits. Graft stiffening and calcification are recognized contributors to late conduit failure after CABG, and strategies that attenuate inflammation-driven remodeling could, in principle, extend graft patency and improve post-operative outcomes. The use of scanning acoustic microscopy to quantify tissue stiffness in a label-free manner, in combination with histological, fluorescence, and elemental analyses, provides a multimodal characterization that strengthens the internal consistency of the observed trends, even in the absence of formal statistical testing. Importantly, the ex vivo, patient-matched design allowed the direct tissue-level effect of L-arginine to be examined while minimizing systemic confounding. Nonetheless, the present observations should be regarded as hypothesis-generating; adequately powered in vivo studies incorporating direct measurements of NO metabolites, inflammatory markers, and longer-term functional endpoints will be required to determine whether L-arginine supplementation can be translated into a clinically meaningful intervention for preserving LIMA graft durability.

In summary, the present study delivers preliminary evidence that L-arginine modulates LIMA graft patency at the histological, morphological, and elemental levels. To consolidate and extend these findings, future studies should recruit larger patient cohorts, incorporate molecular-level mechanistic analyses, and employ more granular specimen subgrouping to ensure sufficient statistical power and interpretive precision.

## 5. Limitations

This study has several limitations that should be considered when interpreting the findings. First, the sample was small (*n* = 18) and comprised exclusively male patients; consequently, the results may not be generalizable to female patients, in whom cardiovascular disease presents differently and perioperative outcomes differ. Second, owing to the limited number of specimens per group, formal statistical analysis was not performed for several endpoints, and the reported differences should be regarded as preliminary and hypothesis-generating rather than confirmatory.

Third, this is an ex vivo model, and the storage and treatment conditions may not fully reproduce the in vivo vascular environment; the model captures selected aspects of inflammation-associated injury but cannot reflect systemic and hemodynamic influences. In addition, a between-graft rather than a within-graft (paired) design was used, since only the short distal LIMA segment normally discarded during graft preparation was available; a within-graft paired design, in which a single segment is divided and exposed to all conditions, would reduce inter-individual variability and is recommended for future studies. Fourth, potential confounders such as hypertension, dyslipidemia, smoking intensity, and genetic background were not measured or adjusted for, which limits causal interpretation of the observed morphological and biomechanical differences. Finally, calcification was assessed at the macroscale; microcalcification was not evaluated. Furthermore, the sample size was not derived from a formal a priori power analysis but was determined by the availability of surplus distal LIMA segments obtained during routine graft preparation; a formal power analysis is recommended to inform the design of future, larger studies. In addition, formal statistical comparison of baseline characteristics (e.g., age, hypertension, hyperlipidemia, and other potential confounders) among the groups and measurement of pre-intervention tissue stiffness were not performed, and such baseline testing is recommended for future, adequately powered studies. These limitations should be addressed in larger, adequately powered, and ideally in vivo studies that include both sexes and detailed clinical characterization.

## 6. Conclusions

This exploratory investigation characterized the tissue-level effects of TNF-α and TNF-α & L-arginine ex vivo conditioning on LIMA grafts from male CABG patients through a multi-modal framework integrating histological staining, Alizarin Red S fluorescence-based calcification detection, SEM/EDS elemental profiling, and SAM-derived biomechanical mapping. Across all modalities, TNF-α treatment consistently produced structural disruption and heightened calcification, whereas L-arginine co-treatment was associated with preservation of organized tissue architecture and diminished calcification-related signatures. Critically, SAM delivered a quantitative mechanical readout through acoustic impedance (Z) mapping, corroborating the interpretation that TNF-α drives LIMA graft stiffening, and that this mechanically detrimental shift is attenuated by L-arginine co-treatment, resulting in partial restoration of graft mechanical integrity. Collectively, these observations indicate that L-arginine may oppose TNF-α-driven remodeling in LIMA grafts and thereby support long-term conduit durability. However, validation in larger, adequately powered cohorts combined with molecular mechanistic studies will be essential before clinical translation can be considered.

## Figures and Tables

**Table 1:** Patient numbers, ages, tissue treatments, and labeling groups of LIMA grafts.

Patient Number	Patient Age (Years)	Tissue Treatment
1	51	Control
2	69	TNF-α
3	70	TNF-α & L-arginine
4	72	Control
5	52	TNF-α
6	74	TNF-α & L-arginine
7	73	Control
8	54	TNF-α
9	70	TNF-α & L-arginine
10	48	Control
11	70	TNF-α
12	65	TNF-α & L-arginine
13	58	TNF-α
14	52	TNF-α & L-arginine
15	68	TNF-α
16	55	TNF-α & L-arginine
17	66	TNF-α
18	53	TNF-α & L-arginine

**Table 2:** Acoustic impedance (Z) values for LIMA grafts. Impedance values are reported in MRayl together with the corresponding measurement uncertainty for each sample.

#Patient	Graft Treatment	Impedance Z (MRayl)	Uncertainty (MRayl)
1	Control	2.20	0.05
2	TNF-α	2.54	0.05
3	TNF-α & L-Arginine	2.17	0.04
4	Control	2.04	0.04
5	TNF-α	2.81	0.05
6	TNF-α & L-Arginine	2.21	0.06
7	Control	2.56	0.09
8	TNF-α	2.76	0.10
9	TNF-α & L-Arginine	2.58	0.05
10	Control	2.23	0.13
11	TNF-α	3.91	0.09
12	TNF-α & L-Arginine	3.59	0.24
13	TNF-α	2.64	0.05
14	TNF-α & L-Arginine	2.29	0.05
15	TNF-α	2.88	0.07
16	TNF-α & L-Arginine	2.60	0.04
17	TNF-α	2.75	0.05
18	TNF-α & L-Arginine	2.10	0.07

**Table 3:** Raw data, atomic percentage (%) results of elements of left internal mammary artery (LIMA) grafts with their uncertainty values obtained by energy dispersive spectroscopy (EDS) analysis.

Patient	Graft	Atomic	Atomic	Atomic	Atomic	Atomic
Number	Treatment	% Carbon	% Nitrogen	% Oxygen	% Phosphorus	% Calcium
3	TNF-α & L-arginine	46.9 ± 0.107	21.4 ± 0.187	26.6 ± 0.141	4.5 ± 0.037	0.6 ± 0.222
3	TNF-α & L-arginine	43.8 ± 0.072	24.9 ± 0.120	29.3 ± 0.112	1.8 ± 0.029	0.3 ± 0.188
3	TNF-α & L-arginine	48.1 ± 0.082	21.7 ± 0.140	27.1 ± 0.120	2.8 ± 0.030	0.2 ± 0.331
4	Control	50.6 ± 0.089	20.9 ± 0.146	24.3 ± 0.122	4.0 ± 0.029	0.2 ± 0.425
4	Control	45.8 ± 0.103	20.7 ± 0.175	28.3 ± 0.131	4.7 ± 0.033	0.5 ± 0.347
4	Control	52.9 ± 0.092	15.1 ± 0.171	26.8 ± 0.118	4.9 ± 0.028	0.2 ± 0.353
8	TNF-α	57.4 ± 0.082	12.7 ± 0.193	26.3 ± 0.121	3.4 ± 0.032	0.3 ± 0.344
8	TNF-α	54.5 ± 0.101	16.6 ± 0.211	23.2 ± 0.141	4.9 ± 0.039	0.7 ± 0.326
8	TNF-α	44.9 ± 0.127	17.6 ± 0.302	32.7 ± 0.170	3.8 ± 0.070	1.0 ± 0.348
10	Control	51.3 ± 0.089	20.1 ± 0.131	22.0 ± 0.114	6.4 ± 0.025	0.3 ± 0.406
10	Control	48.2 ± 0.093	22.8 ± 0.157	23.4 ± 0.131	4.8 ± 0.039	0.9 ± 0.307
10	Control	46.7 ± 0.097	23.1 ± 0.176	26.4 ± 0.139	3.2 ± 0.043	0.5 ± 0.267
11	TNF-α	51.7 ± 0.078	20.8 ± 0.112	22.6 ± 0.105	4.9 ± 0.021	0.1 ± 0.677
11	TNF-α	40.3 ± 0.157	17.3 ± 0.287	32.5 ± 0.160	8.3 ± 0.049	1.6 ± 0.331
11	TNF-α	48.1 ± 0.073	22.4 ± 0.107	26.0 ± 0.102	3.4 ± 0.022	0.1 ± 0.323
14	TNF-α & L-arginine	66.4 ± 0.076	13.8 ± 0.211	16.5 ± 0.147	3.1 ± 0.026	0.2 ± 0.239
14	TNF-α & L-arginine	50.2 ± 0.090	18.2 ± 0.145	26.5 ± 0.117	4.7 ± 0.027	0.3 ± 0.252
14	TNF-α & L-arginine	45.3 ± 0.118	24.0 ± 0.223	26.3 ± 0.173	3.6 ± 0.065	0.8 ± 0.297

## Data Availability

The data presented in this study are available on request from the corresponding author. The data are not publicly available due to privacy and ethical restrictions.
